# American Dental Patents

**Published:** 1853-04

**Authors:** Geo. W. Kendall

**Affiliations:** Cincinnati, Ohio.


### ARTICLE VI.

### *American Dental Patents.* By GEO. W. KENDALL, Cincinnati, Ohio.

SINCE the writing of a sketch of dental patents which was published in the Journal, for April, divers patentees or persons some way connected with patents, have appeared in defence of the propriety of patenting dental improvements; many of their arguments have been ably confuted by Dr. Leslie, but as he has overlooked some of their points, permit me to touch upon them and at the same time continue the "sketch."

As it has been made the subject of invidious remarks that I should have given the patents of Drs. Hill and Allen such particular notice, it may be proper for me to state that my reasons for doing so were that both had advocated the patent system, and they were almost the only dentists whose patents were not abandoned to the profession; they were and are endeavoring by threats and every other means in their power to prevent the profession from using their improvements (?) unless they received a consideration in dollars and cents.

I have seen no cause to change any opinion then advanced, and again reiterate the statements made in regard to the points of the patent law and the validity of the patents noticed.

As an evidence of the upward progress of opinion in reference to the patent system in the profession, we may refer to the resolution passed by the Mississippi Valley Association, at its last meeting, declaring it derogatory to the professional character to patent improvements, the same having been *lost* by an overwhelming vote at the previous meeting.

May we not, therefore, indulge the hope, that ere many years roll around, that instead of a majority of the professors in the "Ohio College of Dental Surgery" advocating by precept or practice the propriety of patenting professional improvements, they will all be united in pointing out to the pupils under their charge the *true* path of the professional gentleman.

It has been urged by our worthy professors that if dentists were not allowed to patent the improvements they might invent or discover, there would be no such improvements announced, as no one would labor to effect them unless he could be *paid for it;* that it is the inalienable right of every man to demand and receive a pecuniary reward for the exercise of the inventive faculties with which he may be endowed, as much as for the labor of his hands. Yet the "disposition to patent every *little* improvement in instruments or appliances for the laboratory is illiberal."

When it comes to the abstract question of individual rights, it would be well for them to remember that the individual has the same *right* to confine the improvement, whether great or small, to his own practice as he has to patent it, and if *money* alone is to be his reward he will most assuredly do so, if he thinks that he can make the most in that way.

And we have the same *right* to patent a compound for destroying nerves, a plugging instrument or a forcep, as we have to patent a dental lathe or mode of making or supplying teeth. With "all due deference to the opinions of others," I think the attempt at making a distinction between those *professional* improvements, which may and which may not be patented, is like

"dividing a hair 'twixt south and southwest side," and it seems the more ludicrous from the fact that those who attempt it are the very ones who think it "the curse of our profession to consider it a mechanical one ! !"

I cannot conceive any position less tenable than that "patenting will cause more improvements to be announced," for notwithstanding the immense number of improvements which have been announced within the last twenty years, scarcely a single one has gone into general use which has been patented, and yet the names of scores of inventors are as familiar as household words. It is also said that a stringent rule against patents would cut off many of the prominent in our profession.

Although it may be very flattering to those few who have taken out patents within the last few years, to be thus ranked among the most eminent, it will be extremely hard for our worthy professor to vindicate their claims to any great eminence, and still more difficult for him to explain, why he makes so great a distinction, between those who use a mode of practice which they choose to keep *secret*, and those who monopolise a mode of practice of equal importance by letters patent. But Dr. Leslie has ably shown up the sophistry, the reasoning, on this point, and so I leave it.

But if your readers will turn to the list of patents in this and the April (1852) number of the Journal, they can decide for themselves, *how* many of the most prominent would be cut off.

It is elsewhere said that we have a United States law, which gives encouragement and protection to the patentee, and gravely asks, "are we prepared to set this law at defiance, by thrusting from us those who seek its protection ?" Let him carry the same spirit a very little further, and he will insist that it is treating the law with *contempt*, to refuse to avail ourselves of its protection in any case where it would apply.

As for a *diversity* of opinion existing among our ablest and best dentists in regard to the propriety of patenting improvements, I can see no evidence of it, but do see those men giving their discoveries to the profession freely and without a thought of peddling them round for a paltry pittance. If that is not positive evidence that the opinion is all on one side I dont know what is. The profession will decide whether it is a breach of good fellowship for a member of a society to receive the improvements which his fellow members may make, and then to turn round and charge them an exorbitant price or even any price for those he makes himself. No, gentlemen, when a man enters our profession or any other, he makes a sacrifice of this much harped on *individual right*, otherwise he is a mere tradesman, and has not the slightest shadow of claim to professional rights.

I dont know which is the most amusing, the hypothesis that a copy-right and a patent-right are identical in principle or the queer gravity with which it is announced and reiterated. It seems to be the main *argument* of those who would defend patents; permit me to refer your readers to the August No. of the Dental Recorder for a notice of Dr. Taylor's remarks in its defence.

The science of dentistry owes its present high position to those noble and generous men who have opened freely their stores of knowledge, actuated by a love of the profession, and an ardent desire for its elevation, considering that superior to all pecuniary considerations, to those "whose eyes even when bent on empty space, beam keen with honor," whose paths are those of the just, and illumined by honors and excellences of an upright, honorable professional life. Our profession like every other, is a unit, and can attain and retain an honorable character only by an uncompromising hostility to the varied forms of empiricism which yet tarnish its fair escutcheon; it does not want nor should it recognize those empirical hangers-on, begot (professionally) of an hermaphrodite, and brought forth in an eclipse

> "Like half-formed insects on the banks of Nile,
> Unfinished things, one knows not what to call
> Their generations so equivocal."

But, enough of this, for why should I continue to argue against the false positions and falser reasoning assumed by these eminent gentlemen? I who am "nothing, a nobody, not even an honorary graduate of the Ohio College of Dental Surgery!"

To the most unanswerable arguments of mine, they would reply with a shrug of affected contempt, some cant phrase or contumelious apothegm, and wrapping themselves up in the dignity acquired by a "practice of many years," whistle reason down the wind, and pocket the dimes.

To you Mr. Editor, and others in high places, must the junior members of the profession look, to have this hydra crushed, do not therefore be content with silent disapproval, but stoop from your lofty station and sweep out this rabble scum.

The following are the only patents for improvements pertaining to dentistry issued since my last article:

*Wm. S. McIlhenney of Philadelphia, March* 23, 1852.

"I claim and desire to secure by letters patent, the formation of artificial teeth, from spar, silex, sand, glass or any other material used for the above purpose into a suitable condition for the finishing furnace by the simple operation of moulding, thereby avoiding the tedious and uncertain process of enameling."

The following are the materials used for composition No. 1:

| | |
|---|---|
| Dis. feldspar, . . | 60 dwt. |
| Feldspar, . . . . | 20 " |
| Quartz, . . . | 20 " |
| White hill clay, . . | 3 " 8 grs. |
| Titanium, . . . . | 12 " |

| | | | | | |
|---|---|---|---|---|---|
| A. | Dis. feldspar, | 2 dwt. | B. | Dis. feldspar, | 4 dwt. |
| | Feldspar, | 2 " | | Feldspar, | 1 " 4 grs. |
| | Quartz, | 1 " | | Quartz, | 1 " 4 " |
| | Brown glass, | ½ " | | White hill clay, | 4 " |

Unite A. and B. in the above proportions for the composition No. 2.

The first process is to place the material while in a plastic state into the respective moulds; the material intended for the back of the tooth (composition, No. 1) is to be worked or packed into that side of the mould.

The portion intended for the face of the tooth (colored composition, No. 2) is to be placed in the same manner and the same condition into the proper side of the mould.

They are then joined and pressed with sufficient force to make the tooth compact, when it is left to dry. When taken from the moulds they are ready for the furnace."

He then goes on and describes the usual mode of manufacture as divided into five heads, viz. moulding, trimming, biscuiting, enameling and trimming the enamel, while he has nothing but the moulding. In "Fitch's Dental Surgery," page 468, the author describes the usual method of enameling teeth, and then says: "in the application of enamel there exists another method which we have equally employed. It is this: make the enamel of the same consistence as the paste, put a certain portion in the bottom of the mould, put again upon the enamel a sufficient quantity of the paste, fill up the mould as usual, &c. The teeth moulded thus, need not undergo the operation of hardening, (biscuiting;) they are then put into a porcelain furnace."

There is precisely the same thing published at least eighteen years ago, and is probably known to all our tooth-makers, and certainly has been adopted to some extent by them.

But it is impossible to make a sufficient variety of teeth to supply the wants of the dentist by this method, and recourse must be had to the secondary operation of enameling. A very handsome tooth is made by using a strongly colored body and putting the enamel into the mould, so that it will be quite thick at the point and gradually diminishing in thickness towards the heel, but the variety is not great.

The patent right to manufacture teeth on this "NEW METHOD," is not worth the paper on which it is written.

*Julius Thompson, North Bridgewater, Massachusetts, September 7th,* 1852.

IMPROVEMENT IN BLOW-PIPES.

What I claim as my invention and desire to secure by letters patent, is

1st. The combination in one instrument of the flame of a lamp or gas, with a blow-pipe, so that both operating together may be held in one hand and the flame applied on any spot, in any direction and for any length of time at the will of the operator.

2nd. The arrangement of a thumb-piece, or its equivalent in combination with a flame of gas or a lamp, and a blow-pipe so that while the instrument is held in one hand a movement of the thumb will adjust the blow-pipe to the flame in such a way as to produce any desired variation in the flame, (blast.)

As an accompaniment to this patent, it may be well to notice a blow-pipe for which an application for a patent has been made, for as much as past experience shows that in matters pertaining to dentistry, a patent will be issued if applied for, no matter how absurd or untenable the claim, we may reasonably suppose that this will soon be on the list of patents, notwithstanding a rumor which now prevails that the application is already rejected.

In the "Dental News Letter," for 1848, was published a description of a blow-pipe invented by Henry Lawrence, which was copied into the "Dental Register," with this remark by the Editor: "The above appears to be (at least to some extent) a description of a blow-pipe, gotten up by *Dr. Van Emon*, of this city." A minute description was published in the Regisister in July, 1849.

Professor Van Emon says, in a letter written since the application for a patent, "the description in the Register is in the main correct, but not accurate enough to enable one to make one that would work well or at all; besides, that would be an infringement which I would not permit. What I claim is, in short, the right of manufacturing and using a bellows constructed on a (the) given plan, or any other that will enable the user to direct in a convenient manner a blast or jet of air, and control it in direction and strength as is done with the mouth blowpipe with as much ease and as quickly. * * * * I charge $25 for them, and require each purchaser to subscribe to the enclosed promise:

"I hereby solemnly promise to use my influence to prevent any infringement on DR. VAN EMON'S BELLOWS and SOLDERING APPARATUS; also, to give him prompt notice of any infringement made on, or about to be made on the same."

"If you sign it, and return it to me with $25, I will send you a bellows, and after using it a month, *if you are not satisfied that it is not what it is represented*, return it and you shall have your money."

This is very fair and modest in *Professor Van Emon*, two qualities which patentees are not prone to.

The whole fixture is a small double bellows, with a flexible tube terminating in a common blow-pipe.

But one case more of a patent applied for, and I will close: Dr. Shephard, a western dentist, has applied for a patent for a mode of putting up teeth, so that the grinding or cutting edges of the teeth shall be covered with metal, obviating the disagreeable sensation sometimes caused by the contact of porcelain teeth. It is done by bringing the strap up over the edge of the tooth, or by soldering the *cap* on to the strap at the corner of the tooth. He was here sometime since offering to sell *rights* to use it.

